# Alterations in intra- and inter-network connectivity associated with cognition impairment in insulinoma patients

**DOI:** 10.3389/fendo.2023.1234921

**Published:** 2023-09-25

**Authors:** Hui Nong, Xiaomin Pang, Jie Jing, Yu Cen, Shanyu Qin, Haixing Jiang

**Affiliations:** ^1^ Department of Gastroenterology, Guangxi Medical University First Affiliated Hospital, Nanning, China; ^2^ Department of Neurology, Guangxi Medical University First Affiliated Hospital, Nanning, China

**Keywords:** insulinoma, cognition impairment, resting-state FMRI, independent component analysis, functional network connectivity

## Abstract

**Objective:**

Cognitive dysfunction is common in insulinoma patients, but the underlying neural mechanisms are less well understood. This study aimed to explore the alterations of intra- and inter-network connectivity patterns associated with patients with insulinoma.

**Methods:**

Resting-state fMRI were acquired from 13 insulinoma patients and 13 matched healthy controls (HCs). Group Independent component analysis (ICA) was employed to capture the resting-state networks (RSNs), then the intra- and inter-network connectivity patterns, were calculated and compared. Montreal Cognitive Assessment (MoCA) was used to assess the cognitive function. The relationship between connectivity patterns and MoCA scores was also examined.

**Results:**

Insulinoma patients performed significantly worse on MoCA compared to HCs. The intra-network connectivity analysis revealed that patients with insulinoma showed decreased connectivity in the left medial superior frontal gyrus within anterior default mode network (aDMN), and decreased connectivity in right lingual gyrus within the visual network (VN). The intra-network connectivity analysis showed that patients with insulinoma had an increased connectivity between the inferior-posterior default mode network (ipDMN) and right frontoparietal network (rFPN) and decreased connectivity between the ipDMN and auditory network (AUN). There was a significant negative correlation between the ipDMN-rFPN connectivity and MoCA score.

**Conclusion:**

This study demonstrated significant abnormalities in the intra- and inter-network connectivity in patients with insulinoma, which may represent the neural mechanisms underlying the cognitive impairment in insulinoma patients.

## 1 Introduction

Insulinomas is a neuroendocrine tumor deriving from β-pancreatic islet cells (1). It is the most common functional neuroendocrine tumor, with an annual incidence of 1 - 4 per million, with a predominance in females (2). As described by Whipple’s triad (3), patients with insulinoma present symptoms and signs of hypoglycemia that are relieved upon normalization of blood glucose levels through glucose administration. The symptomatic episodes can be intermittent, but can become persistent and more frequent as the tumor progresses (4). Since the brain relies almost exclusively on glucose as an energy source, any disturbance in glucose homeostasis may directly affect brain function (5, 6). Several studies have confirmed that hypoglycemia occurs in patients with diabetes mellitus can negatively influence cognitive performance such as learning, memory, and executive function (7–9). In case reports of insulinoma, dementia and transient neurocognitive impairment have also been documented (10, 11). In a recent study conducted by Dai et al., up to 53% of patients with insulinoma were screened positive for cognitive impairment (12). Insulinoma affects cognition across a wide range of functions, including visuospatial and executive functions, delayed memory, attention, language and abstraction (12). These broad cognitive consequences cause a significant decline in the quality of life of patients with insulinoma. Although the cognitive dysfunction exhibited in insulinoma has attracted much attention, the neurobiological mechanisms underlying cognitive impairment remain unclear.

Functional magnetic resonance imaging (fMRI) relies on blood oxygenation level-dependent (BOLD) signal change to indirectly measure neuronal activity, which has become the most prominent tool for the cognitive neuroscience research (13). Previous studies in resting-state fMRI have showed that the intrinsic activity of brain is organized into multiple resting-state networks (RSNs), which appear as groups of anatomically distant but functionally tightly connected brain regions (14, 15). The default mode network (DMN), which is characterized by its activity during rest and subsequent reduction in activity during cognitive tasks, represents one of the most prominent resting-state networks (RSNs) (16). Several other canonical RSNs representing fundamental brain functions, such as sensorimotor network (SMN), visual network (VN), auditory network (AUN), frontoparietal network (FPN), are also widely identified (17). In the past decade, functional connectivity (FC) and functional network connectivity (FNC) methods have been used to explore the dysfunctions of RSNs in a wide range of neurological and neuropsychiatric disorders such as Alzheimer’s disease, Epilepsy, Schizophrenia and Depression, which provide new insights for understanding the pathophysiological mechanisms of cognitive performance in the disease states (18–21).

Currently, the hypoglycemia-induced alterations in connectivity patterns of RSNs have emerged as an interesting topic. An analysis by Nicolas et al. (22) has examined the alterations of RSNs during the induction of hypoglycemia in diabetic and control subjects, and found changes in multiple RSNs, such as DMN, SMN, play an important role in awareness and behavioral response to hypoglycemia. Parikh et al. (23) also found the decreased connectivity in angular gyrus within DMN was correlated with greater symptoms of hypoglycemia as well as higher scores of perceived stresses. Alterations in brain network responses to hypoglycemia are increasingly described, and the brain regions and networks that are sensitive to variations in plasma glucose levels have been identified (24). These abnormalities in brain network have been postulated to contribute to the impaired hypoglycemia awareness or adverse neurocognitive consequences (25, 26). However, the findings mentioned above are derived from patients with diabetes mostly. The alteration of brain network in patients with insulinoma has not been reported.

Independent component analysis (ICA) is one of the most common techniques used to identify RSNs (27). It is a data-driven method that allows the automatic detection and separation of multiple RSNs at once without the need for strict *a priori* hypotheses regarding regions of interest (28). Here, we acquired resting-state fMRI in patients with insulinoma and matched healthy controls (HCs). Group ICA were employed to capture the RSNs, then the FNC, including intra- and inter-network connectivity patterns, were calculated and compared. Also, the Montreal Cognitive Assessment (MoCA) was used to assessed the cognitive function. The relationships between connectivity patterns and MoCA scores were examined. We hypothesize that patients with insulinoma present the abnormal intra- and inter-network connectivity patterns, which are associated with the cognitive impairment.

## 2 Materials and methods

### 2.1 Participants

A total of 13 patients with insulinoma were recruited through the Gastroenterology Department of the First Affiliated Hospital of Guangxi Medical University from January 2022 to December 2022. Initial selection criteria included (1): diagnosed with functional insulinoma (29) (2), age ≥ 18 (3), right-handed. Exclusion criteria were as follows (1): patients with severe underlying diseases, such as severe chronic heart failure and respiratory disorders (2), patients with severe mental or neurological diseases (3), developmental anomalies, cortical malformations, or other focal lesion on structural MRI (4), poor cooperation in the experimental procedures, and (5) contraindications for MRI. Thirteen HCs were recruited from community. They were matched to the patient groups with respect to age, gender, years of education, and handedness. This study was approved by the Medical Research Ethics Committee of the First Affiliated Hospital of Guangxi Medical University, and written informed consent was obtained from each subject.

### 2.2 Cognition assessment

All participants were administered a brief cognitive test using Montreal Cognitive Assessment (MoCA) tool, which has been recommended as a practical and sensitive tool for assessing cognitive impairment (12, 30). We utilized the Beijing version of MoCA. It covers different cognitive domains, including visuospatial and executive functions, naming, memory, attention, language, abstraction, delayed recall, and orientation. The maximum MoCA score is 30 points, and the cutoff point for a screening of mild cognitive impairment is a MoCA score of < 26 (31). The MoCA questionnaire can only be administered when the blood glucose was >2.5 mmol/L in insulinoma patients (12).

### 2.3 MRI data acquisition

MRI data collection and the MoCA evaluation were performed on the same day. An Achieva 3.0 T MRI system scanner (Philips, Amsterdam, The Netherlands) with a standard eight-channel head coil was used to collect imaging data from all participants. The fMRI scan parameters were as follows: repetition time (TR), 2000ms; echo time (TE), 30 ms; flip angle (FA), 90°; field of view, 220 mm × 220 mm; matrix, 64 × 64; in-plane resolution, 3.44 mm × 3.44 mm. For each image volume, 41 transverse slices (slice thickness, 3.5 mm; interslice gap, 0.5 mm) were recorded in an interleaved order, positioned along the line connecting the anterior and posterior commissures (AC-PC orientation), measuring a total of 225 volumes. During scanning, participants were instructed to lie quietly, keep still, keep eyes closed, remain awake, and avoid thinking about anything special. Compliance was confirmed by questioning after the MRI scan. Minimize head movement and scanner noise by using foam pads and earplugs. Other MRI sequences for clinical diagnosis, including T1-weighted imaging (T1WI), T2-weighted imaging (T2WI), and T2 fluid-attenuated inversion recovery (FLAIR), were also acquired but were not used in this study. The schematic illustration of data analysis is shown in **Supplementary Figure 1**.

### 2.4 Data preprocessing

The functional image data were preprocessed using the Data Processing & Analysis for Brain Imaging toolbox (DPABI V6.2, http://www.rfmri.org/dpabi) on the Matrix Laboratory (MATLAB R2013b, https://www.mathworks.com/) platform. For each subject, the first ten volumes of each functional time series were removed to allow stability of the longitudinal magnetization. The remaining images underwent slice-timing correction and realignment, and any subjects with a head movement > 3.0 mm translation or > 3.0° rotation were excluded. Then the functional images were spatially normalized to the Montreal Neurological Institute space and were resampled to a voxel size of 3 mm × 3 mm × 3 mm. The normalized images were spatial smoothed with a 4mm full width at half-maximum Gaussian kernel.

### 2.5 Group ICA and RSNs identification

The spatial group ICA was conducted for the whole subjects using the Group ICA of fMRI Toolbox (GIFT v4.0b, http://icatb.sourceforge.net/). The number of independent components (ICs) was automatically estimated using the minimum description length (MDL) criterion. All the data from the subjects were concatenated into one dataset and reduced in dimensionality using two stages of principal component analysis. The ICs were extracted by the infomax algorithm. This analysis was iterated 20 times using ICASSO to assess the stability and consistency of the extracted components. Then, the time courses and spatial maps were back-reconstructed by group ICA for each participant, and the results were converted to z-scores for display. Among the 20 components arising from ICA, we identified eight meaningful RSNs by applying the previous described procedure (32). Briefly, the spatial map of components should exhibit peak activations in gray matter, no spatial overlap with vascular, ventricular, or susceptibility artefacts. Also, the time courses were dominated by low frequency signal, with the ratio of low-frequency to high-frequency power was greater than 4. These finally determined RSNs were anterior DMN (aDMN), superior-posterior DMN (spDMN), inferior-posterior DMN (ipDMN), right FPN (rFPN), left FPN (lFPN), AUN, SMN, and VN.

### 2.6 Intra-network connectivity analysis

The intra-network connectivity analysis consisted two steps. Firstly, one-sample t tests were conducted for the spatial map of each RSN extracted from ICA of all subjects (patients and HCs). Thresholding was performed with Gaussian random field (GRF) correction at voxel-level *p* < 0.001 and cluster-level *p* < 0.05. The resulting statistical maps were binarized to generate RSN-specific masks. Secondly, group comparisons were carried out for the spatial map of each RSNs using two-sample t-test. Significant voxels were restricted within the respective RNS masks defined in the previous step. Age, sex, and education were taken as nuisance covariates. Correction for multiple comparisons was applied using GRF correction at voxel-level *p* < 0.001 and cluster-level *p* < 0.05.

### 2.7 Inter-network connectivity analysis

Inter-network connectivity was carried out to study the relationship between different RSNs. Before the inter-network connectivity analysis, the time courses of the RSNs of interest were detrended, despiked, and filtered with a high-frequency cutoff of 0.15 Hz (33–35). For each subject, a symmetric 8 × 8 correlation matrix was generated by calculating the Pearson correlation coefficient between the time courses of selected RSNs. The values for the correlation matrixes were transformed to z-scores to improve normality. Group comparisons were conducted using two-sample t-test. Age, sex, and education were taken as nuisance covariates. The significance criterion was p < 0.05 with discovery rate (FDR) for multiple comparisons.

### 2.8 Statistical analysis

The statistical analyses of demographics and MoCA scores were conducted in Statistical Package for the Social Sciences (SPSS V 22.0, www.ibm.com/spss). A normal distribution test was applied to all quantitative data using the Shapiro-Wilk test. To determine the significance of the group differences (patients with insulinoma versus HCs), independent-samples t-test was used for normally distributed data, while the Mann–Whitney U-test was used for non-normally distributed data. Fisher exact probability method was performed to assess the differences in categorical data. Pearson correlation was used to detect associations between the significant differential intra- and inter-network connectivity values and MoCA scores. The significance criterion was *p <*0.05.

## 3 Results

### 3.1 Demographic data and cognitive status

The demographic, clinical and cognitive characteristics of patients with insulinoma and HCs are summarized in **Table 1**. No significant differences in age, gender, and education level were found between the groups (*p* > 0.05). As expected, patients with insulinoma performed significantly worse on the MoCA (*p* < 0.05). Among patients with insulinoma, eight (61%) patients presented with cognitive impairment. Further analysis of the MoCA sub-domains revealed that cognitive performance was particularly affected in visuospatial and executive functions, naming, attention, language, memory, and delayed recall in patients with insulinoma.

**Table 1:** Demographic, clinical characteristics and cognitive performance of the patients with insulinoma and HCs.

	Insulinoma patients (n=13)	HCs (n=13)	t/Z value	*p* value
Demographic characteristics				
Age (years)	50.77 ± 16.04	45.38 ± 9.93	-1.029	0.314^a^
Sex (male/female)	1/12	3/10	/	0.593^b^
Education level (years)	9.31 ± 4.94	11.38 ± 3.73	1.210	0.238^a^
Clinical characteristics				
BMI (kg/m^2^)	23.93 ± 3.61	/	/	/
Duration of disease (months)	83.96 ± 75.11	/	/	/
Tumor size (cm)	1.13 ± 0.47	/	/	/
Lowest blood glucose (mmol/L)	1.70 ± 0.40	/	/	/
MoCA score	25(17~27)	30(27~30)	-3.139	0.002^c^
MoCA subdomain scores				
Visuospatial/executive	4(3~5)	5(4.5~5)	-2.303	0.021 ^c^
Naming	2(1~3)	3(3~3)	-2.579	0.010 ^c^
Attention	5(4~6)	6(6~6)	-2.419	0.016 ^c^
Language	2(0.5~2)	3(2.5~3)	-3.352	0.001 ^c^
Abstraction	2(0.5~2)	2(2~2)	-0.976	0.329 ^c^
Memory and delayed recall	3(1~4)	5(3.5~5)	-2.682	0.007 ^c^
Orientation	6(4.5~6)	6(6~6)	-1.184	0.236 ^c^

Continuous data are presented as the mean ± SD or median [interquartile range (IQR)]. For all tests, p < 0.05 was considered statistically significant. ^a^p values were obtained by two-sample t-test. ^b^p values were obtained by Fisher exact probability method. ^c^p values were obtained by two-tail Mann-Whitney U test.

HCs, healthy controls; BMI, body mass index; MoCA, Montreal Cognitive Assessment.

### 3.2 RSNs identification

**Figure 1:**
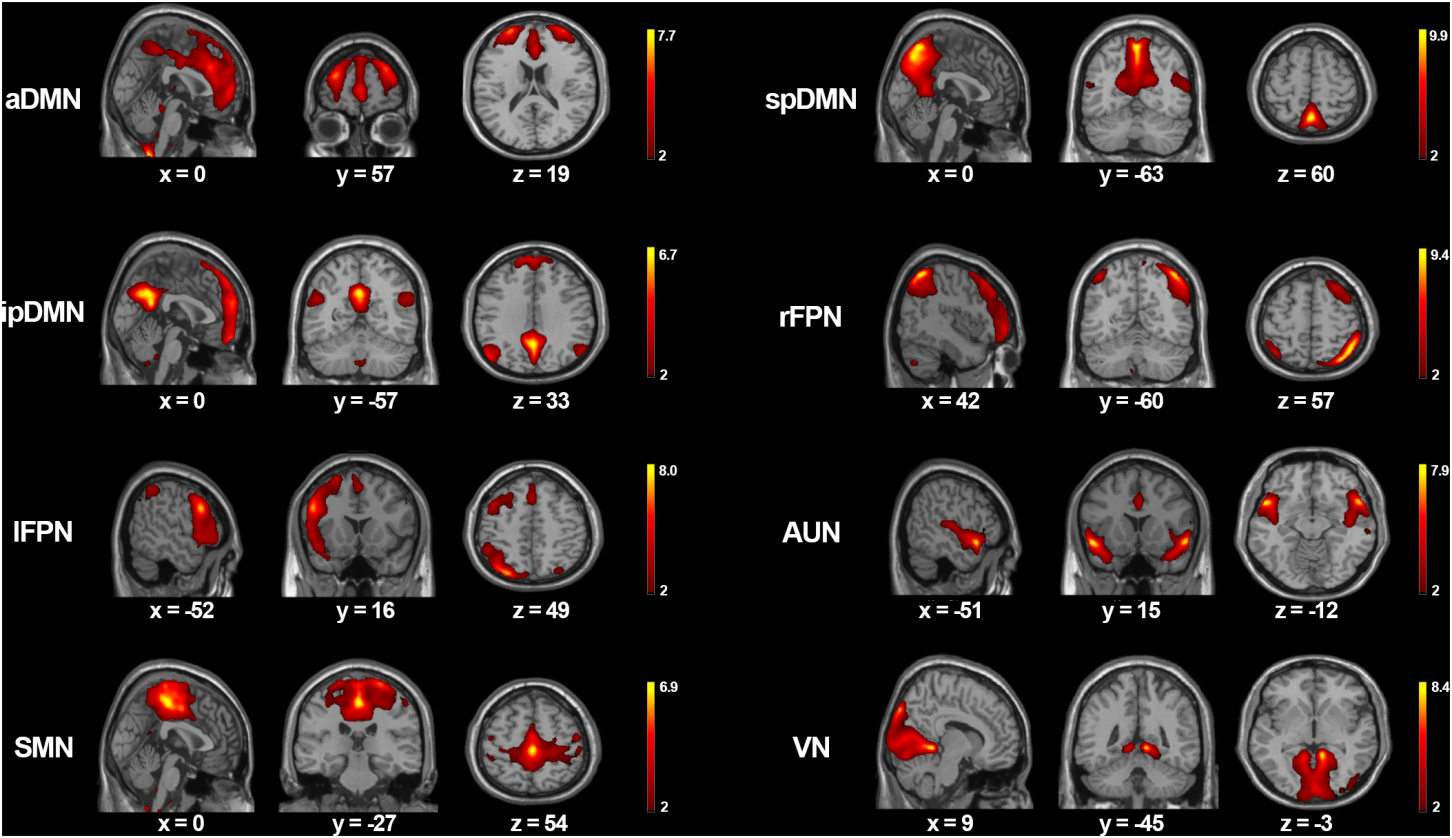
The spatial maps of eight meaningful RSNs. Each RSNs was thresholded at z > 2 and the MNI coordinates of the peak values are displayed in three orthogonal planes. aDMN, anterior default mode network; spDMN, superior-posterior default mode networ; ipDMN, inferior-posterior default mode network; rFPN, right frontoparietal network; lFPN, left frontoparietal network; AUN, auditory network; SMN, sensorimotor network; VN, visual network.

Spatial maps of the eight functionally relevant RSNs are presented in **Figure 1**. In our analysis, the DMN comprised three components, i.e., the aDMN with a large cluster in bilateral medial prefrontal cortex and anterior cingulate cortex, the spDMN included primarily bilateral precuneus and posterior cingulate cortex, and the ipDMN consisted of bilateral precuneus and angular gyrus. Regarding the two FPN components, the right FPN (rFPN) were comprised primarily of right dorsolateral prefrontal cortex and inferior parietal lobule, while the left FPN (lFPN) were comprised primarily of left dorsolateral prefrontal cortex and inferior parietal lobule. The AUN mainly consisted of bilateral superior temporal gyrus and middle temporal gyrus. The SMN was mostly composed of bilateral precentral gyrus, postcentral gyrus and paracentral lobule. The VN primarily contains bilateral cuneus, lingual gyrus and middle occipital gyrus.

### 3.3 Group comparisons of intra-network connectivity

**Figure 2:**
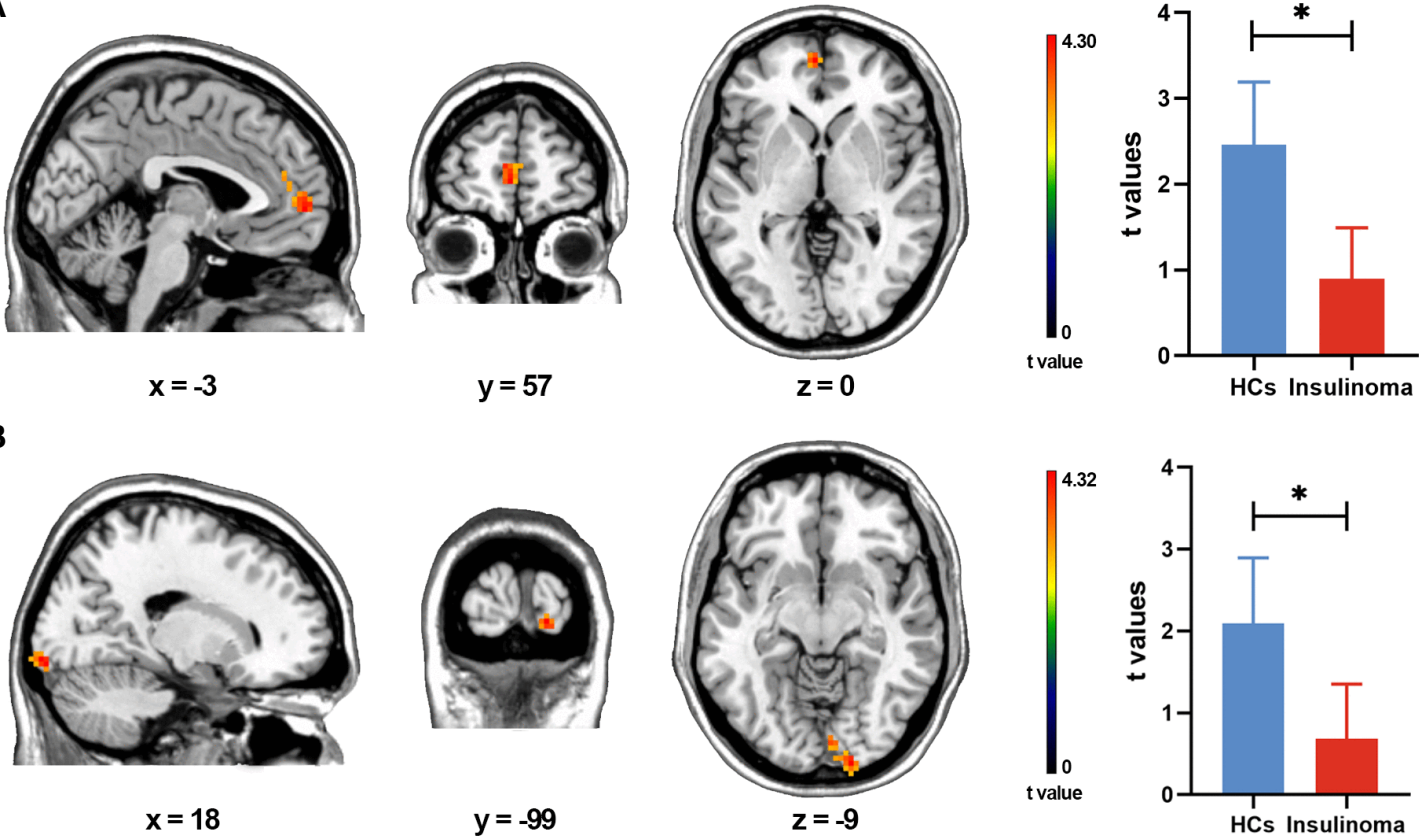
Intra-network connectivity analysis of the patients with insulinoma and HCs. Group differences in intra-network connectivity within the aDMN **(A)** and VN **(B)** were obtained by two-sample t-test (GRF correction, voxel-level *p* < 0.001 and cluster-level *p* < 0.05). The extracted t-values for the significant brain regions across all subjects were compared and subsequently presented in the form of a bar plot (*p < 0.05).

Group comparisons of intra-network connectivity are displayed in **Table 2** and **Figure 2**. Significant connectivity differences within aDMN and VN were found between the HCs and insulinoma patients (GRF correction, voxel-level *p* < 0.001 and cluster-level *p* < 0.05). Compared to HCs, insulinoma patients with insulinoma showed decreased connectivity in the left medial superior frontal gyrus within aDMN, and decreased connectivity in right lingual gyrus within the VN. There was no significant alteration in connectivity within other RSNs between the groups (*p* > 0.05).

**Table 2:** Group differences in intra-network connectivity between the patients with insulinoma and HCs.

RSNs	Brain regions (AAL)	Voxels	Peak coordinates (MNI)			Peak t values
			x	y	z	
aDMN	Frontal_Sup_Medial_L	41	-3	57	0	4.2984
VN	Lingual_R	34	18	-99	-9	4.3237

Results were obtained by two-sample t-test (GRF correction, voxel-level p < 0.001 and cluster-level p < 0.05). Brain regions were determined by AAL atlas. Peak coordinates are reported in MNI space.

RSNs, resting-state networks; AAL, anatomical automatic labeling; MNI, Montreal Neurological Institute; L, left; R, right.

### 3.4 Group comparisons of inter-network connectivity

**Figure 3:**
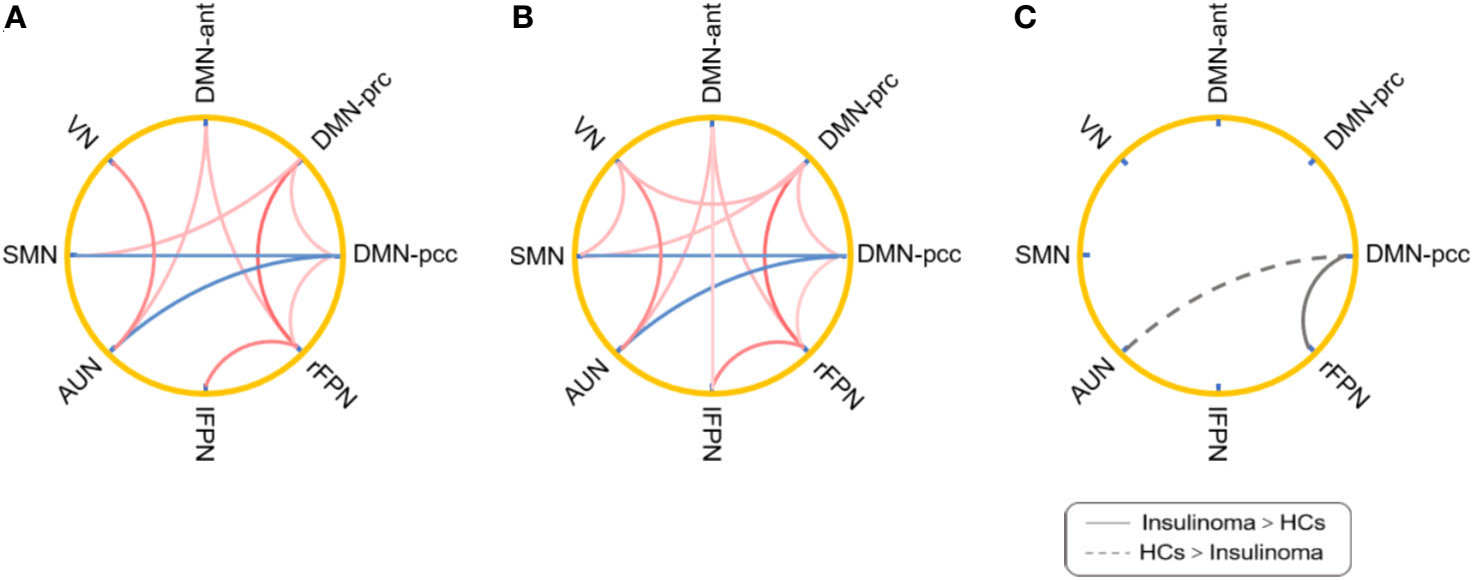
Inter-network connectivity analysis of the patients with insulinoma and HCs. The inter-network functional connectivity patterns for the patients with insulinoma **(A)** and HC **(B)** were obtained by one-sample t-test (*p* < 0.05, uncorrected). The red lines represent positive connections and blue lines denote negative connections. Group differences of inter-network connectivity **(C)** were obtained by two-sample t-test (*p* < 0.05, uncorrected). The solid lines represent the increased connections and dotted lines represent decreased connections.

Group comparisons of inter-network connectivity are displayed in **Figure 3**. Significant connectivity differences between the ipDMN and rFPN, and between the ipDMN and AUN were found between the HCs and insulinoma patients (*p* < 0.05, uncorrected). Compared to HCs, patients with insulinoma showed increased connectivity between the ipDMN and rFPN and decreased connectivity between the ipDMN and AUN. There was no significant alteration in connectivity between other RSNs between the groups (*p* > 0.05).

### 3.5 Correlation analysis

**Figure 4:**
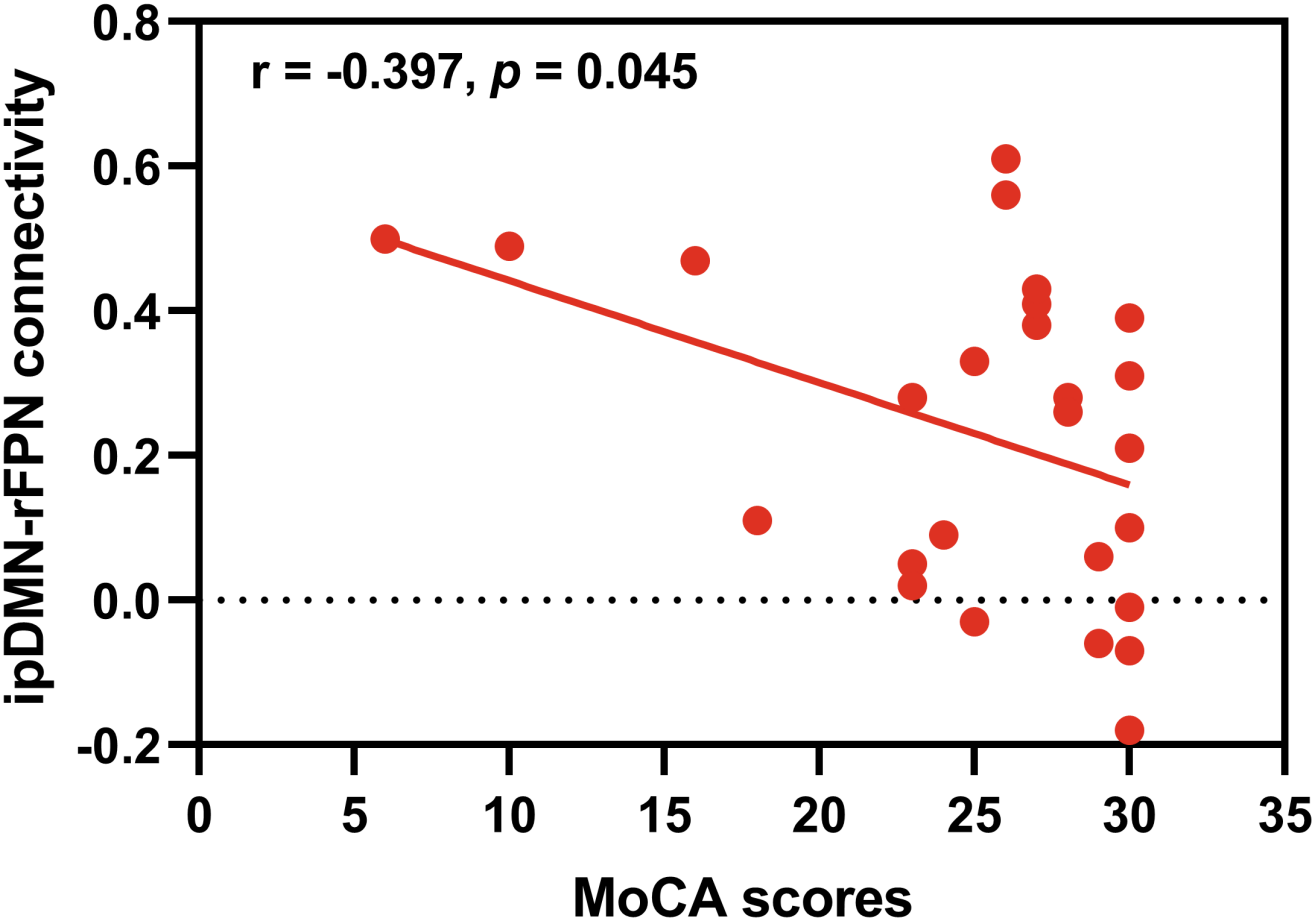
Correlation analysis. Significant negative correlation was found between the ipDMN-rFPN connection and MoCA scores (*p* < 0.05, Bonferroni correction).

Correlation analyses were performed to investigate the relationships between the significant differential intra- and inter-network connectivity values and MoCA scores. As showed in **Figure 4**, significant negative correlation was found between the ipDMN-rFPN connectivity and MoCA scores (*p* < 0.05, Bonferroni correction). There was no significant correlation between the ipDMN-AUN connection and MoCA scores (*p* > 0.05). No significant correlation was found between the intra-network connectivity and MoCA scores (*p* > 0.05).

## 4 Discussion

Cognitive dysfunction is common in insulinoma patients, but the underlying neural mechanisms are less well understood. To the best of our knowledge, this is the first exploratory study to characterize the functional network connectivity patterns and their relation to cognitive function in insulinoma patients, imaged in a non-hypoglycemic state. Several findings emerged (1). Patients with insulinoma showed cognitive impairment across a wide range of functions (2). Patients with insulinoma presented decreased connectivity within the aDMN and the VN, as well as increased connectivity between the ipDMN and rFPN and decreased connectivity between the ipDMN and AUN (3). The connectivity between the ipDMN and rFPN were associated with worsening cognitive performance. These observations are particularly important for understanding the neural mechanisms of cognitive impairment in insulinoma patients.

In this study, we observe that 61% (8/13) of insulinoma patients show cognitive dysfunction, which is consistent with the previous study (12). Specifically, we found that significant impairments in several cognitive domains, such as visuospatial and executive functions, memory, attention, naming and language, were visible in insulinoma patients compared to the HCs. Although no significant statistical difference was found in other cognitive domains, a decreasing trend could still be found. This would seem to suggest that insulinoma may induce cognitive impairment in multiple domains, which may require a more detailed cognitive scale to confirm.

To explore the neural mechanism of the cognitive impairment in insulinoma patients, we calculate and compare the intra- and inter-network connectivity patterns between insulinoma patients and HCs. The most important results of the present study are the differences of intra-network connectivity within the DMN. The DMN is one of the most robust RSNs, reflecting intrinsic brain activity in a default state (36). Because it was first discovered through its stronger activity at rest compared to task performance, early DMN functionality was focused on associations with internally generated cognition, including daydreaming, mind wandering, or self-related thought. Subsequent findings suggest a role for the DMN in external as well as internal focused cognition (37). Anatomically, the DMN consists of a set of distributed regions including the medial prefrontal cortex, the posterior cingulate cortex and the adjacent precuneus plus the lateral parietal cortex (38). The medial prefrontal cortex is a key region of the DMN involved in numerous cognitive functions, including attention, inhibitory control, habit formation and working, and spatial or long-term memory (39). A growing body of literature suggests that decreased connectivity within the DMN has been observed in individuals with multiple neuropsychiatric conditions with cognitive impairment, such as Alzheimer’s disease, schizophrenia, Parkinson’s disease, depression, and anxiety, particularly in the medial prefrontal cortex (40, 41). In the current study, the patients with insulinoma presented a decreased connectivity within the aDMN, predominantly located in the left medial superior frontal gyrus. This disrupted DMN pattern may be key to cognitive dysfunction in insulinoma patients. On the other hand, it is interesting to note that DMN has a high metabolically active nature, which makes it preferentially more susceptible to altered glucose levels than other neural networks (42). A study by Susanne has demonstrated a close relationship between local glucose consumption and DMN functional connectivity (43). More recently, alterations in functional connectivity within the DMN in response to hypoglycemia have increasingly been described in patients with diabetes mellitus (25). The DMN dysfunction we describe in the current study may also reflect the effect of hypoglycemia on neural integrity in patients with insulinoma.

Existing evidences have been demonstrated that hypoglycemia reduce visual acuity (44). A meta-analysis involving 1657 infants found that neonatal hypoglycemia was associated with an increased risk of poor visual motor function (45). A study conducted by *Valente et al.* has observed that visual function impairment was present in 24 of 54 patients with endogenous hyperinsulinemia hypoglycemia (including 40 insulinoma patients) (46). These phenomena may be due to the fact that the retina, one of the most metabolically active tissues, requires a constant supply of glucose to maintain optimal function (47, 48). Another explanation is that hypoglycemia may induce acute injury of the occipital lobes, resulting in cortical visual dysfunction (49). The specific pattern of injury in neonatal hypoglycemia, with the occipital lobes being most severely affected, has been documented in several imaging studies (50, 51). The fact that the occipital cortex is so sensitive to hypoglycemia is presumably a result of the hypoperfusion and excitatory toxicity of this region in association with cell-type specific injury (49, 50). In the presented study, we found a decrease in connectivity in the occipital region within the VN in patients with insulinoma. The VN is located bilaterally in the occipital cortex and extends into the temporo-occipital junction (52), which plays a primary role in visual information processing (53). This finding here reflects an underlying visual dysfunction in insulinoma patients, which is consistent with previous studies. Clearly, visual impairment in insulinoma deserves more particular attention in the future.

The human brain is a highly efficient integrative network that links sub-networks together into a complex system in which information is continuously processed and transported (21). Functional communication among RSNs is likely to play a key role in maintaining the complex cognitive processes in the human brain (54). In this way, we further explore the interaction patterns of RSNs in patients with insulinoma. Our results reveal that patients with insulinoma show increased ipDMN-rFPN connectivity and decreased ipDMN-AUN connectivity compared to HCs. The DMN and FPN operate in opposite fashion in the brain, representing a task-negative network and a task-positive network, respectively. The interaction between DMN and FPN is more cooperative than competitive (55, 56), and contributes to the completion of tasks (56, 57), memory search (58), and faster memory retrieval (59). Aberrant connectivity between two networks is often indicative of disruption of well-organized cognitive control processes, which may well head to widespread cognitive dysfunction (60, 61). The pattern of increased ipDMN-rFPN connectivity we observe here reflects less efficient cognitive processing in patients with insulinoma. Notably, we also observed a negative correlation between the ipDMN-rFPN connectivity and MoCA score in the current study, suggesting that hyperconnectivity is associated with poorer cognitive performance. We speculate that the increased ipDMN-rFPN connectivity is a potential compensation mechanism for maintaining cognitive function in insulinoma patients with inefficient information processing in the brain. As for the decreased ipDMN-AUN connectivity, this suggests that the brain allocates more neural resources to cope with the decline in higher cognitive functions, resulting in less resources available for primary perceptual functions such as hearing (60).

There are also some limitations that should be considered. First, due to the relatively small sample size, the results have to be viewed with caution. Future studies with larger sample sizes are required. Second, although this study was performed using strict inclusion and exclusion criteria, the effect of heterogeneity remains. Some of the patients had received endoscopic treatment for insulinoma, but the rest had not. More subgroups and a longitudinal study design would be appropriate. Finally, our results do not reflect the features of temporal variability within brain networks, and therefore dynamic FNC is considered as a future research direction.

## 5 Conclusion

In summary, this study suggests an anomalous connectivity of intra- and internetwork in patients with insulinoma, which may represent the neural mechanism underlying the cognitive impairment in patients with insulinoma.

## Data availability statement

The raw data supporting the conclusions of this article will be made available by the authors, without undue reservation.

## Ethics statement

The studies involving humans were approved by Medical Research Ethics Committee of the First Affiliated Hospital of Guangxi Medical University. The studies were conducted in accordance with the local legislation and institutional requirements. The participants provided their written informed consent to participate in this study.

## Author contributions

SQ and HJ contributed to conception and design of the study. HN wrote the first draft of the manuscript. JJ and YC collected clinical and MRI data. HN and XP analyzed the MRI data. All authors contributed to the article and approved the submitted version.
