# Supplementary material for: Alterations in intra- and inter-network connectivity associated with cognition impairment in insulinoma patients
Source: Front Endocrinol (Lausanne). 2023 Sep 25;14:1234921. doi: 10.3389/fendo.2023.1234921 (PMC10561291; doi:10.3389/fendo.2023.1234921)
Supplement: Supplementary file 1 [file Image_1.pdf]

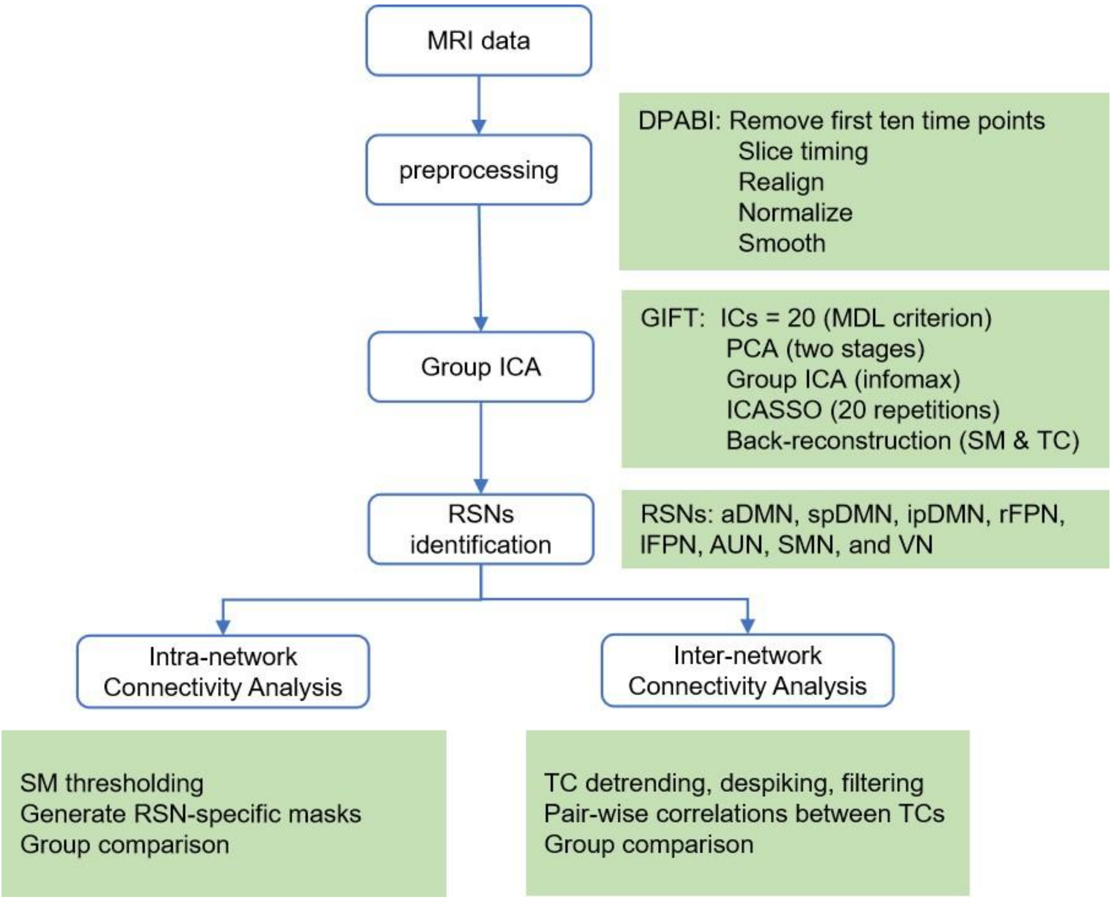

Supplementary Figure 1. The schematic illustration of data analysis. ICs, independent components; MDL, the minimum description length; PCA, principal component analysis; SM, spatial maps; TC, time courses.
